# Development and Optimization of In-house ELISA for Detection of Human IgG Antibody to SARS-CoV-2 Full Length Spike Protein

**DOI:** 10.3390/pathogens9100803

**Published:** 2020-09-28

**Authors:** Thamir A. Alandijany, Sherif A. El-Kafrawy, Ahmed M. Tolah, Sayed S. Sohrab, Arwa A. Faizo, Ahmed M. Hassan, Tagreed L. Alsubhi, Norah A. Othman, Esam I. Azhar

**Affiliations:** 1 Special Infectious Agents Unit, King Fahd Medical Research Center, King Abdulaziz University, P.O. Box 128442, Jeddah 21362, Saudi Arabia; saelkfrawy@kau.edu.sa (S.A.E.-K.); atoulah@kau.edu.sa (A.M.T.); ssohrab@kau.edu.sa (S.S.S.); aafaizo@kau.edu.sa (A.A.F.); hmsahmed@kau.edu.sa (A.M.H.); tlalsobhe@kau.edu.sa (T.L.A.); naothman@kau.edu.sa (N.A.O.); eazhar@kau.edu.sa (E.I.A.); 2 Department of Medical Laboratory Technology, Faculty of Applied Medical Sciences, King Abdulaziz University, P.O. Box 80324, Jeddah 21589, Saudi Arabia

**Keywords:** COVID-19, SARS-CoV-2, ELISA, immunoassay

## Abstract

The ongoing coronavirus disease 19 (COVID-19) pandemic, caused by the novel severe acute respiratory syndrome coronavirus 2 (SARS-CoV-2), poses a threat to human health. Despite this, many affected countries are now in the process of gradual lifting of COVID-19 restrictions that were initially implemented in response to the pandemic. The success of the so-called “exit strategy” requires continued surveillance of virus circulation in the community and evaluation of the prevalence of protective immunity among population. Serology tests are valuable tools for these purposes. Herein, SARS-CoV-2 full-length spike (S) recombinant protein was utilized to develop and optimize an indirect enzyme-linked immunoassay (ELISA) that enables a reliable detection of virus-specific IgG antibody in human sera. Importantly, the performance of this assay was evaluated utilizing micro-neutralization (MN) assay as a reference test. Our developed ELISA offers 100% sensitivity, 98.4% specificity, 98.8% agreement, and high overall accuracy. Moreover, the optical density (OD) values of positive samples significantly correlated with their MN titers. The assay specifically detects human IgG antibodies directed against SARS-CoV-2, but not those to Middle East respiratory syndrome coronavirus (MERS-CoV) or human coronavirus HKU1 (HCoV-HKU1). The availability of this in-house ELISA protocol would be valuable for various diagnostic and epidemiological applications.

## 1. Introduction

The coronavirus disease 2019 (COVID-19) is a major public health issue caused by the novel severe acute respiratory syndrome coronavirus 2 (SARS-CoV-2) [1]. It was declared a pandemic by the World Health Organization (WHO) on 11 March 2020 [2]. COVID-19 patients present varied clinical features that range from no or mild symptoms to severe life-threatening manifestations [3]. Pulmonary and extra-pulmonary complications can lead to death of COVID-19 patients [4,5,6]. The main routes of viral transmission include respiratory droplets and direct contact with infected individuals [7,8]. The potential of aerosol transmission has also been reported [9]. Importantly, it has been suggested that asymptomatic COVID-19 cases can be infectious to healthy persons, which may have significantly contributed to the massive spread of the infection [7].

Although officials in the affected countries have implemented various strategies to control the infection, SARS-CoV-2 infection will likely continue to spread due to the lack of effective vaccines or treatments [10,11]. Furthermore, many countries are in the process of gradual lifting of COVID-19 restrictions. During this transition phase and through the post-pandemic period, conducting mass screening can play a significant role in infection control and prevention. It enables a comprehensive understanding of the disease status, and allows identification and isolation of contagious individuals [12,13,14]. Several factors should be considered before choosing the test(s) to be utilized for mass screening. These include cost, turnaround time, sensitivity, specificity, throughput, and required infrastructure setting in addition to the type of results obtained from these tests (e.g., quantitative vs. qualitative, direct detection of viral antigens/nucleic acids vs. indirect detection of infection-specific antibodies).

Currently, the gold standard method for COVID-19 diagnosis is reverse transcriptase polymerase chain reaction (RT-PCR) utilizing primer/probe sets, which allows detection of the viral nucleocapsid (N), RNA-dependent RNA polymerase (RdRP), and envelope (E) genes [15]. Factors like viral load, sample collection and transportation, RNA extraction protocol, type of enzyme inhibitors, and RT-PCR method are crucial when performing RT-PCR to avoid false negative results [16,17]. Traditional viral culture is valuable, but it is time-consuming and requires biosafety level-3 facilities that are not widely available [18]. Although their usage remains limited, novel technologies such as clustered regularly interspersed short palindromic repeats (CRISPR) and loop-mediated isothermal amplification (LAMP) have been utilized to develop rapid and efficient diagnostic tests [19,20]. Serology testing is a reliable, simple and cost-effective technique that allows direct and indirect detection of infections. Historically, it has been efficiently applied for epidemiological surveillance studies. Further, several serology-based rapid point-of-care tests have been developed to reduce the assay turnaround time [21].

In this study, we have developed an enzyme-linked immunoassay (ELISA) based on SARS-CoV-2 full-length spike (S) protein that enables sensitive and specific detection of virus-specific IgG antibody in human sera. To our knowledge, this is the first in-house ELISA that is evaluated against micro-neutralization (MN) assay (the gold standard to assess the presence of virus-specific antibodies and measure their neutralization). In addition to its potential diagnostic application, the availability of this assay could be valuable to gain knowledge on the seroprevalence status of COVID-19 in population, which is vitally important for disease control and prevention.

## 2. Results

### 2.1. Sero-Status of Samples

The total number of samples in this study was 418 human sera collected from healthy persons, healthcare workers, COVID-19 patients, and recovered individuals. SARS-CoV-2 MN assay was conducted to determine the sero-status of these samples with MN titer of ≥ 1:20 considered positive. The numbers of negative and positive samples were 309 and 109, respectively.

### 2.2. Optimization of ELISA Protocol

**Figure 1:**
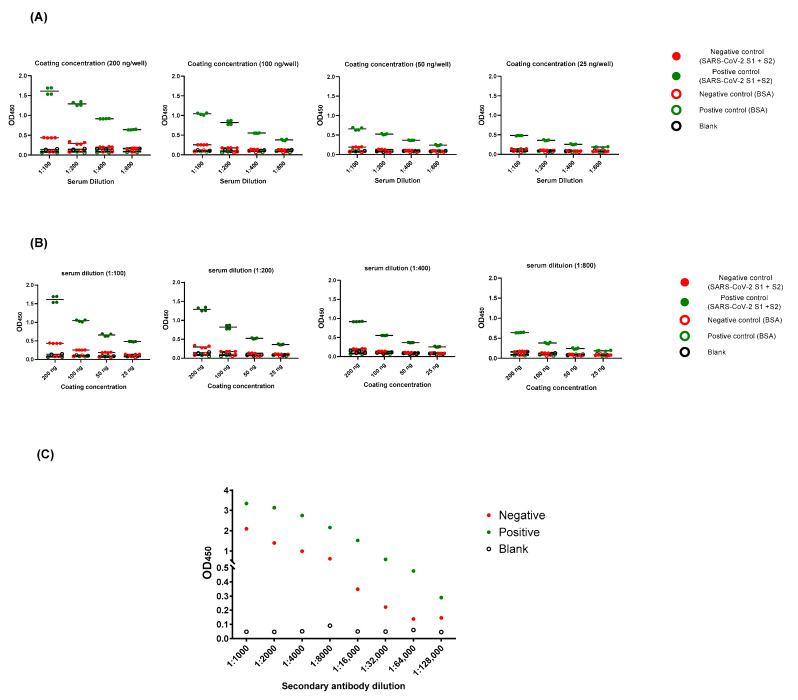
Optimization of an indirect ELISA utilizing SARS-CoV-2 full-length S (S1 + S2) recombinant protein. (**A**) and (**B**) Plates were coated overnight at 4 ℃ with different concentrations of either SARS-CoV-2 full-length S (S1 + S2) extracellular domain with a polyhistidine tag (ECD-His) recombinant protein, or bovine serum albumin (BSA) as a coating control. Following three washes with PBS containing 0.1% Tween 20 (PBST), positive (green) and negative (red) samples based on micro-neutralization assay were serially diluted (as indicated) and added. PBST was used as blank (empty black circles). Following an hour of incubation at 37 ℃, three washes with PBST were performed followed by incubation with conjugate (peroxidase-labelled anti-human IgG secondary antibody) for 1 h at 37 ℃. Then, three washes with PBST were performed followed by the addition of 3,3’,5,5’-Tetramethylbenzidine (TMB) substrate for 5 min. Hydrochloric acid (HCL) was added as a stop solution. The optical density was measured at 450 nm (OD_450_). (**C**) Plates were coated with 100 ng/well of viral antigen, samples were used at 1:100 dilution, and conjugate dilutions ranged from 1:1000 to 1:128,000. The protocol was performed as described above.

In order to optimize the assay condition, it is important to provide a protocol that offers sufficient antigen to capture antibody, but avoids high non-specific backgrounds. Hence, plates were initially coated with SARS-CoV-2 full length S (S1 + S2) ECD-His recombinant protein at a range of concentrations (from 25 to 200 ng per well), serum samples were serially diluted (from 1:100 to 1:800), and the conjugate was used at different dilutions (1:1000 to 1:128,000) (Figure 1)**.** Two elements determined our optimized condition: (1) the highest OD_450_ ratio of positive to negative samples, and (2) the lowest OD_450_ ratio between negative samples to blank. Optimized working condition was as follows: 100 ng per well for coating, 1:100 of sample dilution, and 1:64,000 of conjugate dilution. This protocol was used for all subsequent experiments.

### 2.3. Cut-off Value and Evaluation of the Developed ELISA

**Figure 2:**
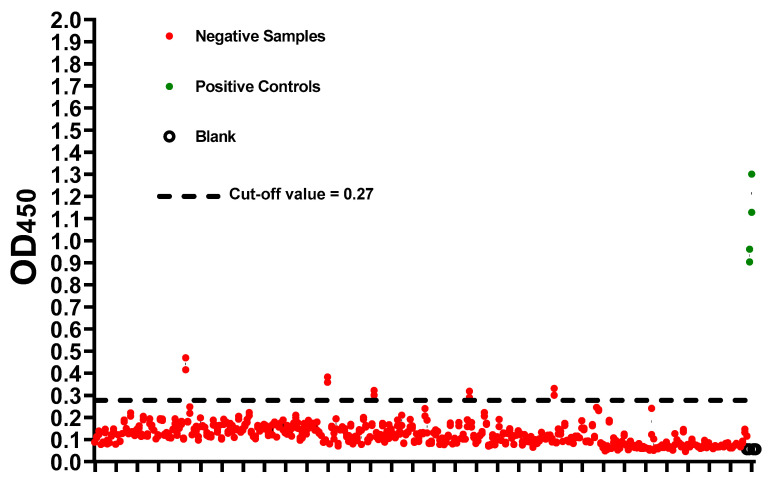
The cut-off value of the developed SARS-CoV-2 full-length S (S1 + S2)-based indirect ELISA. Plates were coated overnight at 4 ℃ with 100 ng/well of SARS-CoV-2 full-length S (S1 + S2) ECD-His recombinant protein. Following three washes with PBS containing 0.1% Tween 20 (PBST), positive (green) and negative (red) samples based on micro-neutralization assay were diluted 1:100 and added. PBST was added as blank (empty circles). Following an hour of incubation at 37 ℃, three washes with PBST were performed followed by incubation with the conjugate (peroxidase-labelled anti-human IgG secondary antibody at 1:64,000 dilution) for 1 h at 37 ℃. Then, three washes with PBST were performed followed by the addition of 3,3’,5,5’-Tetramethylbenzidine (TMB) substrate for 5 min. Hydrochloric acid (HCL) was added as a stop solution. The optical density was measured at 450 nm (OD_450_). The actual values for each sample is shown. Dashed lines represent the cut-off value (Mean + 3 x standard deviation).

**Figure 3:**
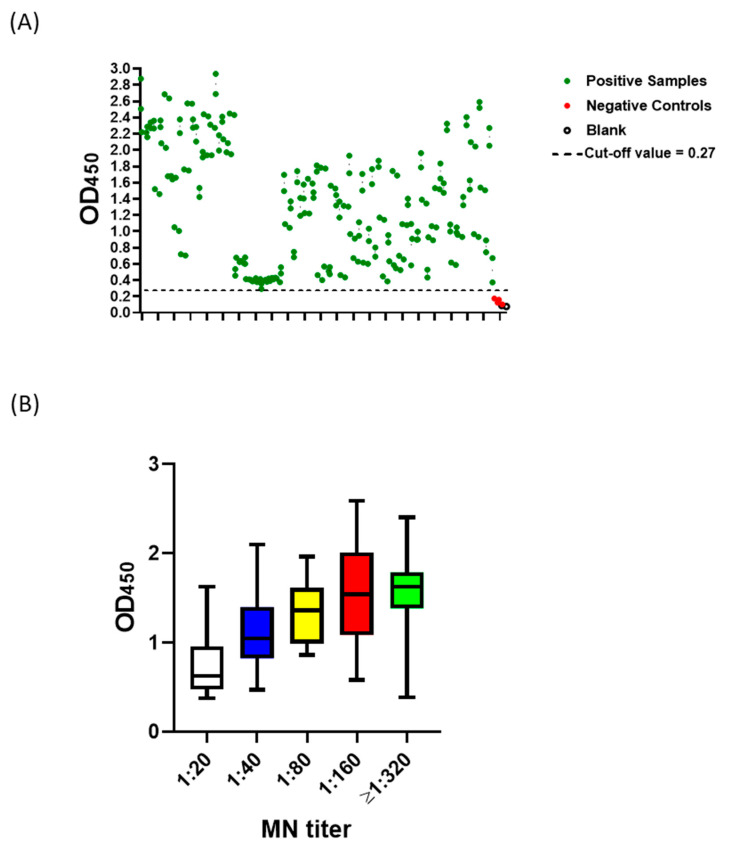
Evaluation of the developed SARS-CoV-2 full-length S (S1 + S2)-based indirect ELISA. Plates were coated overnight at 4 ℃ with 100 ng/well of SARS-CoV-2 full-length S (S1 + S2) ECD-His recombinant protein. Following three washes with PBST, positive (green) and negative (red) samples based on micro-neutralization assay were diluted 1:100 and added. PBS containing 0.1% Tween 20 (PBST) was added as blank (empty circles). Following an hour of incubation at 37 ℃, three washes with PBST were performed followed by incubation with the conjugate (peroxidase-labelled anti-human IgG secondary antibody at 1:64,000 dilution) for 1 h at 37 ℃. Then, three washes with PBST was performed followed by the addition of 3,3’,5,5’-Tetramethylbenzidine (TMB) substrate for 5 min. Hydrochloric acid (HCL) was added as a stop solution. The optical density was measured at 450 nm (OD_450_). Dashed lines represent the cut-off value (Mean + 3 x standard deviation). **(A)** shows the actual values for each sample, and **(B)** demonstrates the correlation between OD_450_ readings and micro-neutralization (MN) titer. Boxes show the 25th–75th percentile range, the black line represents median, and whiskers are the minimum and maximum values.

**Figure 4:**
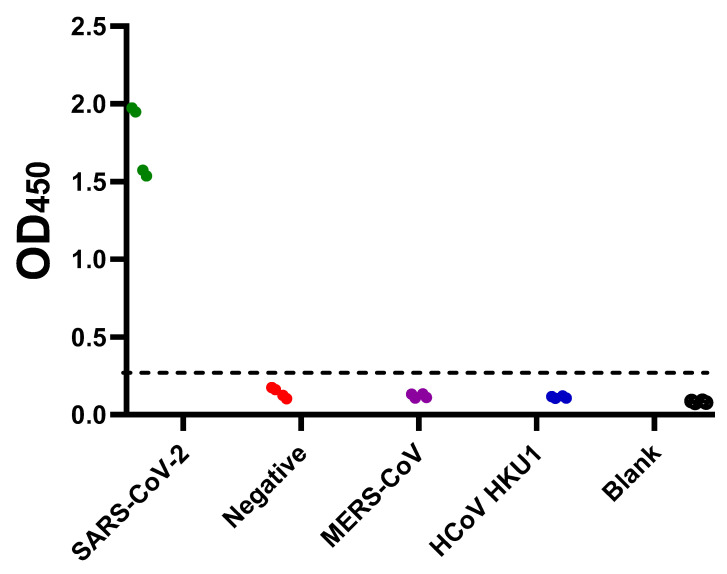
Assessment of the cross-reactivity of the developed SARS-CoV-2 S1 + S2 indirect ELISA. Plates were coated overnight at 4 ℃ with 100 ng/well of SARS-CoV-2 full-length S (S1 + S2) ECD-His recombinant protein. Following three washes with PBST, Serum samples containing anti-SARS-CoV-2 antibodies (green), anti-Middle East respiratory syndrome coronavirus (MERS-CoV) antibodies (purple), and anti-human coronavirus HKU1 (HCoV HKU1) antibodies (blue) were diluted 1:100 and added. Serum from healthy donors were used as a negative control (red). PBS containing 0.1% Tween 20 (PBST) was added as blank (empty circles). Following an hour of incubation at 37 ℃, three washes with PBST were performed followed by incubation with the conjugate (peroxidase-labelled anti-human IgG secondary antibody at 1:64,000 dilution) for 1 h at 37 ℃. Then, three washes with PBST were performed followed by the addition of 3,3’,5,5’-Tetramethylbenzidine (TMB) substrate for 5 min. Hydrochloric acid (HCL) was added as a stop solution. The optical density was measured at 450 nm (OD_450_).

Negative samples (n = 309) based on MN assay were used to determine the preliminary cut-off value, which was calculated as mean + (3 x standard deviation). The preliminary cut-off value of this developed ELISA was 0.27. Apart from five samples, OD_450_ readings of negative samples (MN titer <1:20) were below the preliminary cut-off value (Figure 2). On the other hand, all positive samples (n = 109) predetermined with MN assay were tested positive (OD_450_ > 0.27) (Figure 3A). Moreover, a statistically significant correlation between OD_450_ values and MN titers was observed (r_s_ = 1, *p* value = 0.016) (Figure 3B)**.** Similar results were obtained from three independent experiments. Assessment of inter-assay and intra-assay variation demonstrated high reproducibility with <12% variation. Collectively, these data demonstrate 100% sensitivity and 98.4% specificity with 98.8% agreement of our developed ELISA. Importantly, this assay is specific for the detection of human IgG antibody directed against SARS-CoV-2, as sera containing Middle East respiratory syndrome coronavirus (MERS-CoV) or (MERS-CoV) or human coronavirus HKU1 (HCoV HKU1) antibodies tested negative (Figure 4).

**Figure 5:**
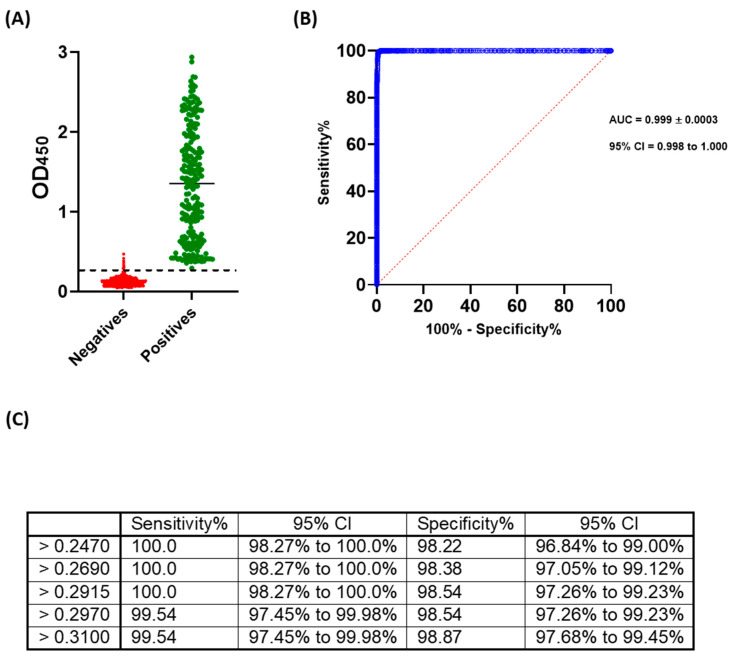
Receiver operating characteristics (ROC) analysis of the developed ELISA. ROC analysis was performed for positive samples based on results obtained from micro-neutralization assay. (**A**) shows the distribution of data utilized for ROS analysis, (**B**) demonstrates ROC curve, and (**C**) presents a range of cut-off values and their associated sensitivity and specificity with 95% confidence interval (CI) as obtained from ROC analysis.

Next, ROC analysis was performed in order to assess the accuracy and determine the cut-off value that offers maximum sensitivity and specificity of this assay. The developed and optimized ELISA demonstrated high accuracy with area under curve (AUC) equal to 0.9996 ± 0.0003; 95% confidence interval of 0.99 to 1.00. As per analysis, the cut-off value that provides 100% sensitivity and 98.54% specificity is 0.29. A range of OD_450_ readings and their associated sensitivity and specificity percentages are shown (Figure 5).

Collectively, data presented demonstrate that the described ELISA protocol in this study provides a valid protocol that enables sensitive and specific detection of anti-SARS-CoV-2 specific IgG antibody in human sera.

## 3. Discussion

SARS-CoV-2 has continued to massively spread around the globe posing a threat to human populations [22]. Despite early control measures (e.g., travel restriction, social distancing, and educational institutes and places of worship closure), officials in many affected countries have decided to gradually lift these restrictions and relax regulations [10,12,23]. Active surveillance, isolation of new cases and clusters, and estimation of seroprevalence among populations are required in order to succeed in this “back-to-normal” plan [24]. Serology-based techniques are particularly useful for large scale and high throughput screening. They do not require biosafety containment level 3 facilities or highly specialized equipment. Moreover, it is usually easy to develop serology-based rapid point-of-care kits. Many commercial kits are already available. However, the performance of these kits remains uncertain [25,26,27]. Results, in terms of sensitivity and specificity, necessitate proper validation prior to utilizing them for diagnostic and epidemiological applications [25,26,27]. A recent evaluation of six commercial COVID-19 serology kits that enable detection of IgG either individually or in combination with IgM demonstrated a range of sensitivity from 81% to 100% and specificity from 85% to 99% [26]. An independent systematic review and meta-analysis of the performance of several commercial and in-house SARS-CoV-2 antibody tests revealed 66.7% to 97.9% sensitivity and 88.8% to 100% specificity [25]. Hence, there is a need for the development of reliable serology testing for both diagnostic and epidemiological purposes.

Here, we have developed and optimized an in-house ELISA protocol based on the SARS-CoV-2 full-length S (S1 + S2) protein that enables the detection of viral-specific human IgG. Importantly, we have evaluated the sensitivity and specificity of our protocol against MN assay, which represents the gold standard for evaluating antibody-mediated protective immunity against SARS-CoV-2 [25,26,27].

Our optimized condition involves coating plates with 100 ng per well of antigen, screening samples at 1:100 dilution, and using 100 µL of 1:68,000 conjugate dilution. Higher coating, sample, or conjugate concentration increased non-specific backgrounds (Figure 1). The preliminary cut-off value was 0.27, which was determined as the mean of negative samples + (3 x standard deviation) (Figure 2). The OD_450_ values of all positive samples (MN titer ≥ 1:20) were > 0.27 (Figure 3A). Moreover, these values positively correlated with MN titers (Figure 3B). Apart from five false positive samples, OD_450_ values of all negatives samples (MN titer < 1:20) were below 0.27 (Figure 2). Our developed ELISA offers 100% sensitivity, 98.4% specificity, 98.8% agreement, and high accuracy of with AUC of 0.9996 ± 0.0003 (95% confidence interval of 0.99 to 1.00) (Figure 5). Further, it does not cross react with sera containing antibodies to other relative coronaviruses (MERS-CoV and HCoV HKU1) (Figure 4). We believe that the described ELISA protocol can be deployed for both diagnostic and screening purposes.

## 4. Materials and Methods

### 4.1. Cell Line and Virus

African green monkey kidney cells Vero E6 (ATCC^®^ CRL-1586™) were grown and maintained at 37 ℃ in 5% CO2. The local SARS-CoV-2 clinical isolate (SARS-CoV-2/human/SAU/85791C/2020) (Gene accession number MT630432.1) was propagated and titrated by Median Tissue Culture Infectious Dose (TCID50).

### 4.2. Samples

Serum samples of healthy individuals, healthcare workers, COVID-19 patients, and recovered individuals (n = 418) were utilized in this study. Their sero-status as positive or negative for SARS-CoV-2 antibody were determined based on the micro-neutralization (MN) assay performed in biosafety-containment level 3 laboratory located at the special infectious agents unit (SIAU), King Fahd Medical Research Centre (KFMRC), King Abdulaziz University (KAU). Samples containing Middle East respiratory syndrome coronavirus (MERS-CoV) and human coronavirus (HCoV) HKU1 antibodies were used as specificity controls.

### 4.3. Micro-Neutralization (MN) Assay

Sera were heat inactivated at 56 ℃ for 30 min, serially diluted in, and added with equal volume of Dulbecco’s Modified Eagle Medium (DMEM) containing 100 TCID50 of SARS-CoV-2 on confluent Vero E6 cells. Then, cells were incubated at 37 ℃ in 5% CO2 for 3–4 days. SARS-CoV-2 infected cells in the absence of human serum and uninfected cells were used as positive and negative controls, respectively. MN titers were determined as the highest dilution of serum sample that utterly prevented the viral-induced cytopathic effect with MN titer of ≥ 1:20 considered positive.

### 4.4. Development and Optimization of Indirect ELISAs

SARS-CoV-2 (2019-nCoV) spike S1 + S2 ECD-His recombinant protein (Sino Biological, Beijing, China) was utilized for ELISA development. Bovine serum albumin (BSA) (Thermo Fisher Scientific, Waltham, Massachusetts, USA) was used as a coating control. Positive (n = 109) and negative (n = 309) samples for SARS-CoV-2 antibodies based on MN assay were utilized for ELISA optimization. Samples containing MERS-CoV and HCoV HKU1 antibodies were used for cross-reactivity evaluation.

Flat Bottom Microtiter plates (Immulon^®^ 2 HB, Bloomington, Minnesota, USA) were coated overnight at 4 ℃ with 100 ng per well of recombinant proteins diluted in phosphate buffer saline (PBS). The plates were washed three times with PBS containing 0.1% Tween 20 (PBST), blocked in 5% skimmed milk for 1 h at room temperature followed by three washes with PBST. Samples were prepared at 1:100 dilutions in 5% skimmed milk and added at 100 µL volume per well. After an hour of incubation at 37 ℃, wells were washed three times with PBST. Conjugate (goat KPL peroxidase-labelled antibodies to human IgG; Seracare, Milford, Massachusetts, USA) at a dilution of 1:64,000 in PBST were added at 100 µL volume per well and incubated for an hour at 37 ℃. Following three washes with PBST, 100 µL of 3,3’,5,5’-Tetramethylbenzidine (TMB) (Seracare, Milford, Massachusetts, USA) were added for 5 min for color development. The reaction was stopped by the addition of 100 µL of 1 N hydrochloric acid (HCL). The absorbance was read at 450 nm using Elx 800 bioelisa Reader (Biokit, Barcelona, Spain).

### 4.5. Statistical Analyses

The cut-off values were determined as follows: Mean of negative controls+(3 × standard deviation). The sensitivity was calculated as:

(1) $$\left(\frac{\mathrm{the}\;\mathrm{number}\;\mathrm{of}\;\mathrm{true}\;\mathrm{positive}}{\mathrm{the}\;\mathrm{total}\;\mathrm{number}\;\mathrm{of}\;\mathrm{true}\;\mathrm{positive}+\;\mathrm{false}\;\mathrm{negative}\;\mathrm{samples}}\right)\times \;100$$

The specificity was calculated as:

(2) $$\left(\frac{\mathrm{the}\;\mathrm{number}\;\mathrm{of}\;\mathrm{true}\;\mathrm{negative}}{\mathrm{the}\;\mathrm{total}\;\mathrm{number}\;\mathrm{of}\;\mathrm{true}\;\mathrm{negative}+\;\mathrm{false}\;\mathrm{positive}\;\mathrm{samples}}\right)\times \;100$$

The agreement was calculated as:

(3) $$\left(\frac{\mathrm{the}\;\mathrm{total}\;\mathrm{number}\;\mathrm{of}\;\mathrm{true}\;\mathrm{positive}+\mathrm{true}\;\mathrm{negative}\;\mathrm{samples}}{\mathrm{the}\;\mathrm{total}\;\mathrm{number}\;\mathrm{of}\;\mathrm{samples}}\right)\times \;100$$

The correlation between the optical density at 450 nm (OD_450_) values and MN titer was assessed by Spearman’s rank-order correlation with *p* value < 0.05 considered statistically significant. The receiver-operating characteristic (ROC) was calculated to determine the threshold value that separates positive from negative taking into consideration the sensitivity and specificity percentages of the assay. Inter-assay and intra-assay variation was assessed to evaluate the reproducibility of the assay. Figure drawing and data processing were performed by GraphPad Prism software utilizing MN assay as a reference test.

### 4.6. Ethic Statement

This study was approved by the Research Ethics Committee (REC), Unit of Biomedical Ethics, Faculty of Medicine, King Abdulaziz University (Reference No: 487-20). Written consent forms were obtained from participants.

## 5. Conclusions

Serology techniques such as ELISA are valuable tools for patients’ diagnosis and population screening. However, it is crucial to utilize a valid protocol in order to obtain reliable results. The ELISA protocol described in this study enables sensitive, specific, and reliable detection of human anti-SARS-CoV-2 IgG antibody. Having a valid in-house ELISA for COVID-19 is beneficial as many countries are currently in the transition phase to “return-to-normal” life.

## References

[1] World Health Organization (WHO) (2020). Director-General’s Remarks at the Media Briefing on 2019-nCoV on 11 February 2020. https://www.who.int/dg/speeches/detail/who-director-general-s-remarks-at-the-media-briefing-on-2019-ncov-on-11-february-2020.

[2] World Health Organization (WHO) (2020). Director-General’s Opening Remarks at the Media Briefing on COVID-19-11 March 2020. https://www.who.int/dg/speeches/detail/who-director-general-s-opening-remarks-at-the-media-briefing-on-covid-19---11-march-2020.

[3] Chan K.W., Wong V.T., Tang S.C.W. (2020). COVID-19: An Update on the Epidemiological, Clinical, Preventive and Therapeutic Evidence and Guidelines of Integrative Chinese-Western Medicine for the Management of 2019 Novel Coronavirus Disease. Am. J. Chin. Med..

[4] Gupta A., Madhavan M.V., Sehgal K., Nair N., Mahajan S., Sehrawat T.S., Bikdeli B., Ahluwalia N., Ausiello J.C., Wan E.Y. (2020). Extrapulmonary manifestations of COVID-19. Nat. Med..

[5] Zhou F., Yu T., Du R., Fan G., Liu Y., Liu Z., Xiang J., Wang Y., Song B., Gu X. (2020). Clinical course and risk factors for mortality of adult inpatients with COVID-19 in Wuhan, China: A retrospective cohort study. Lancet.

[6] Leung C. (2020). Clinical features of deaths in the novel coronavirus epidemic in China. Rev. Med. Virol..

[7] Arons M.M., Hatfield K.M., Reddy S.C., Kimball A., James A., Jacobs J.R., Taylor J., Spicer K., Bardossy A.C., Oakley L.P. (2020). Presymptomatic SARS-CoV-2 Infections and Transmission in a Skilled Nursing Facility. N. Engl. J. Med..

[8] Chan J.F.-W., Yuan S., Kok K.-H., To K.K.-W., Chu H., Yang J., Xing F., Liu J., Yip C.C.-Y., Poon R.W.-S. (2020). A familial cluster of pneumonia associated with the 2019 novel coronavirus indicating person-to-person transmission: A study of a family cluster. Lancet.

[9] Meselson M. (2020). Droplets and Aerosols in the Transmission of SARS-CoV-2. N. Engl. J. Med..

[10] Alandijany T.A., Faizo A.A., Azhar E.I. (2020). Coronavirus disease of 2019 (COVID-19) in the Gulf Cooperation Council (GCC) countries: Current status and management practices. J. Infect. Public Health.

[11] Chaudhry R., Dranitsaris G., Mubashir T., Bartoszko J., Riazi S. (2020). A country level analysis measuring the impact of government actions, country preparedness and socioeconomic factors on COVID-19 mortality and related health outcomes. EClinicalMedicine.

[12] Peto J. (2020). Covid-19 mass testing facilities could end the epidemic rapidly. BMJ (Clin. Res. Ed.).

[13] Kwon K.T., Ko J.H., Shin H., Sung M., Kim J.Y. (2020). Drive-Through Screening Center for COVID-19: A Safe and Efficient Screening System against Massive Community Outbreak. J. Korean Med. Sci..

[14] Sunjaya A.F., Sunjaya A.P. (2020). Pooled Testing for Expanding COVID-19 Mass Surveillance. Disaster Med. Public Health Prep..

[15] Pascarella G., Strumia A., Piliego C., Bruno F., Del Buono R., Costa F., Scarlata S., Agrò F.E. (2020). COVID-19 diagnosis and management: A comprehensive review. J. Intern. Med..

[16] Kucirka L.M., Lauer S.A., Laeyendecker O., Boon D., Lessler J. (2020). Variation in False-Negative Rate of Reverse Transcriptase Polymerase Chain Reaction–Based SARS-CoV-2 Tests by Time Since Exposure. Ann. Intern. Med..

[17] Woloshin S., Patel N., Kesselheim A.S. (2020). False Negative Tests for SARS-CoV-2 Infection—Challenges and Implications. N. Engl. J. Med..

[18] Bain W., Lee J.S., Watson A.M., Stitt-Fischer M.S. (2020). Practical Guidelines for Collection, Manipulation and Inactivation of SARS-CoV-2 and COVID-19 Clinical Specimens. Curr. Protoc. Cytom..

[19] Broughton J.P., Deng X., Yu G., Fasching C.L., Servellita V., Singh J., Miao X., Streithorst J.A., Granados A., Sotomayor-Gonzalez A. (2020). CRISPR–Cas12-based detection of SARS-CoV-2. Nat. Biotechnol..

[20] Augustine R., Hasan A., Das S., Ahmed R., Mori Y., Notomi T., Kevadiya B.D., S Thakor A. (2020). Loop-Mediated Isothermal Amplification (LAMP): A Rapid, Sensitive, Specific, and Cost-Effective Point-of-Care Test for Coronaviruses in the Context of COVID-19 Pandemic. Biology.

[21] Peeling R.W., Wedderburn C.J., Garcia P.J., Boeras D., Fongwen N., Nkengasong J., Sall A., Tanuri A., Heymann D.L. (2020). Serology testing in the COVID-19 pandemic response. Lancet Infect. Dis..

[22] World Health Organization (WHO) (2020). COVID-19 Dashboard. https://who.sprinklr.com.

[23] Wu Z., McGoogan J.M. (2020). Characteristics of and Important Lessons from the Coronavirus Disease 2019 (COVID-19) Outbreak in China: Summary of a Report of 72314 Cases From the Chinese Center for Disease Control and Prevention. JAMA.

[24] Kissler S.M., Tedijanto C., Goldstein E., Grad Y.H., Lipsitch M. (2020). Projecting the transmission dynamics of SARS-CoV-2 through the postpandemic period. Science.

[25] Lisboa Bastos M., Tavaziva G., Abidi S.K., Campbell J.R., Haraoui L.-P., Johnston J.C., Lan Z., Law S., MacLean E., Trajman A. (2020). Diagnostic accuracy of serological tests for covid-19: Systematic review and meta-analysis. BMJ (Clin. Res. Ed.).

[26] GeurtsvanKessel C.H., Okba N.M.A., Igloi Z., Bogers S., Embregts C.W.E., Laksono B.M., Leijten L., Rokx C., Rijnders B., Rahamat-Langendoen J. (2020). An evaluation of COVID-19 serological assays informs future diagnostics and exposure assessment. Nat. Commun..

[27] Kohmer N., Westhaus S., Rühl C., Ciesek S., Rabenau H.F. (2020). Clinical performance of different SARS-CoV-2 IgG antibody tests. J. Med. Virol..

